# High-Throughput Screening for Bacterial Glycosyltransferase Inhibitors

**DOI:** 10.3389/fcimb.2018.00435

**Published:** 2018-12-18

**Authors:** Samir El Qaidi, Congrui Zhu, Peter McDonald, Anuradha Roy, Pradip Kumar Maity, Digamber Rane, Chamani Perera, Philip R. Hardwidge

**Affiliations:** ^1^College of Veterinary Medicine, Kansas State University, Manhattan, KS, United States; ^2^High Throughput Screening Laboratory, University of Kansas, Lawrence, KS, United States; ^3^Synthetic Chemical Biology Core Laboratory, University of Kansas, Lawrence, KS, United States

**Keywords:** bacterial pathogenesis, glycosylteransferase, innate immnuity, signal transduction, virulence, type III secreted effector protein

## Abstract

The enteropathogenic and enterohemorrhagic *Escherichia coli* NleB proteins as well as the *Salmonella enterica* SseK proteins are type III secretion system effectors that function as glycosyltransferase enzymes to post-translationally modify host substrates on arginine residues. This modification is unusual because it occurs on the guanidinium groups of arginines, which are poor nucleophiles, and is distinct from the activity of the mammalian *O*-linked *N*-acetylglucosaminyltransferase. We conducted high-throughput screening assays to identify small molecules that inhibit NleB/SseK activity. Two compounds, 100066N and 102644N, both significantly inhibited NleB1, SseK1, and SseK2 activities. Addition of these compounds to cultured mammalian cells was sufficient to inhibit NleB1 glycosylation of the tumor necrosis factor receptor type 1-associated DEATH domain protein. These compounds were also capable of inhibiting *Salmonella enterica* strain ATCC 14028 replication in mouse macrophage-like cells. Neither inhibitor was significantly toxic to mammalian cells, nor showed *in vitro* cross-reactivity with the mammalian *O*-linked *N*-acetylglucosaminyltransferase. These compounds or derivatives generated from medicinal chemistry refinements may have utility as a potential alternative therapeutic strategy to antibiotics or as reagents to further the study of bacterial glycosyltransferases.

## Introduction

Many Gram-negative bacterial pathogens interact with mammalian cells by using a specialized type III secretion system (T3SS) to inject proteins directly into infected host cells (Notti and Stebbins, 2016; Deng et al., 2017). A subset of these injected protein “effectors” are enzymes that modify the structure and function of human proteins by catalyzing the addition of unusual post-translational modifications (Ribet and Cossart, 2010; Salomon and Orth, 2013). Some T3SS effectors play important roles in bacterial virulence and may be “druggable” targets for anti-virulence compounds that could be used to replace or augment traditional antibiotic regimens (Kimura et al., 2011; Warrener et al., 2014; Gu et al., 2015; McShan and De Guzman, 2015).

The NleB1/NleB2 (enteropathogenic and enterohemorrhagic *E. coli*; EPEC and EHEC) and SseK1/SseK2/SseK3 (*Salmonella enterica*) T3SS effectors are glycosyltransferases that modify protein substrates on arginine residues (Li et al., 2013; Pearson et al., 2013). This modification is unusual because it occurs on the guanidinium groups of arginines, which are poor nucleophiles. This modification is biologically important because the glycosylation of arginines on protein substrates disrupts the normal functioning of the innate immune system (Li et al., 2013; Pearson et al., 2013; El Qaidi et al., 2017). Several death domain-containing proteins have been described as substrates of some of the NleB/SseK orthologs, including the Fas-associated protein with death domain (FADD), tumor necrosis factor receptor type 1-associated DEATH domain protein (TRADD), and the receptor-interacting serine/threonine-protein kinase 1 (RIPK1) (Li et al., 2013). Glyceraldehyde 3-phosphate dehydrogenase (GAPDH) (Gao et al., 2013) and hypoxia-inducible factor 1-alpha (HIF1α) (Xu et al., 2018) are additional substrates. Recent structural characterization of a subset of the NleB/SseK orthologs revealed that these proteins are retaining glycosyltransferases consisting of three functional domains, namely a helix-loop-helix (HLH) domain, a lid domain, and a catalytic domain with a HEN motif (His-Glu-Asn) that is required for catalytic activity (Esposito et al., 2018; Park et al., 2018).

NleB1 is a signature of EHEC strains that have been observed to cause widespread foodborne outbreaks that develop into cases of hemolytic uremic syndrome (HUS) (Wickham et al., 2006). HUS, which is caused by the activity of a Shiga-like toxin, leads to destruction of the endothelial cells of the kidney and is often fatal (Loos et al., 2018). Antibiotic therapy against EHEC is typically contraindicated, because antibiotics may induce increased Shiga-like toxin expression, exacerbating patient symptoms (Wong et al., 2000). This motivated our work, which sought to identify small molecule inhibitors of NleB1 as a potential alternative therapeutic strategy to antibiotics. Such compounds may also be of utility as mechanistic probes for understanding the mechanism of the NleB/SseK effector family.

## Results

### NleB1 Inhibitor Screening

**Figure 1:**
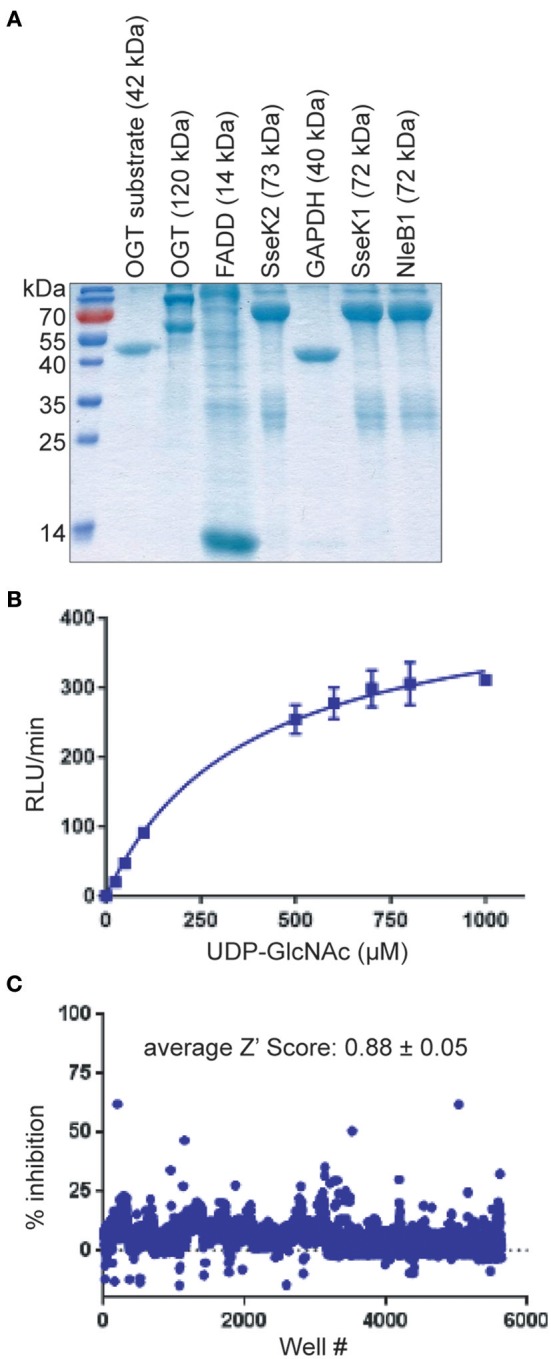
HTS assay. **(A)** SDS-PAGE analysis of the recombinant proteins used in this study. **(B)** UDP liberation (RLU/min) is plotted as a function of substrate (UDP-GlcNAc) concentration in the presence of NleB1. **(C)** Summary of HTS assay data. The % inhibition of UDP liberation is plotted as a function of the well number of each compound represented in the CMLD library of 5,160 compounds. Compounds (*n* = 52) that inhibited the assay to >3 standard deviations ± the plate median were identified as positive hits.

We developed and optimized a high-throughput screening (HTS) assay for NleB1 inhibitors. Purified, recombinant forms of wild-type *E. coli* O157:H7 NleB1 (Figure 1A) and, as a negative control, an inactive form of NleB1 (NleB1-AAA) in which the aspartic acid residues required for Mn^2+^ stabilization were mutated to alanines, were produced (Gao et al., 2013). We then optimized the conditions in which these enzymes could be studied in a solution-based assay that produces a luminescent signal when UDP-GlcNAc is hydrolyzed by NleB1 to liberate UDP (Figure 1B).

Optimal NleB1 activity was observed at 0–25 mM NaCl and 10 mM MgCl_2_, with >50% loss of activity at 500 mM NaCl and at 50 mM MgCl_2_. NleB1 activity increased linearly up to 2 h and no loss of activity was observed up to 4% DMSO. The kinetic parameters of NleB1 (150 nM at 30°C) were calculated as follows: Vmax: 2,975.3 ± 125 RLU/min/μg protein; Km: 379 ± 43 μM; Kcat (s-1): 50, Kcat/Km (s-1, M-1): 130,703.4. As expected, the NleB1-AAA mutant enzyme had no detectable activity (*not shown*).

We then screened the University of Kansas Center of Excellence in Chemical Methodologies and Library Development (*KU CMLD*, https://cmld.ku.edu/outreach) library of 5,160 compounds. This library utilizes new principles of scaffold design, incorporates multiple scaffold cores into a single library, and contains compounds that are likely to have pharmacological activity based on sound drug design principles. The structures and bioactivity data of these compounds are described in *PubChem* (https://www.ncbi.nlm.nih.gov/pcsubstance/?term=%22ku+outreach+library%2C+the+university+of+kansas%22%5Bsourcename%5D).

**Figure 2:**
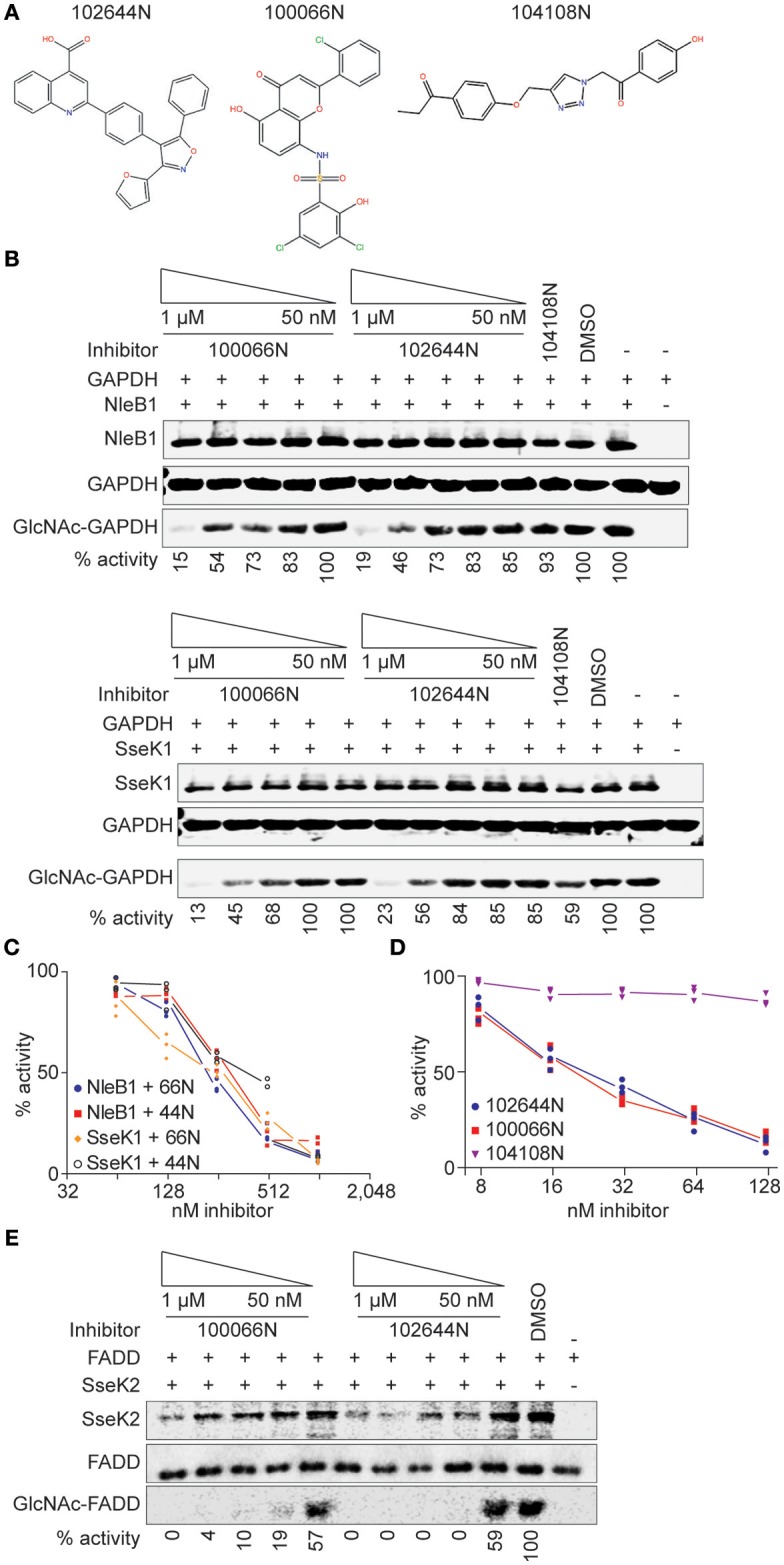
*In vitro* glycosylation assays. **(A)** 100066N, 102644N, and 104108N structures. **(B)** Western blot analysis of the inhibition of NleB1 and SseK1 glycosylation of GAPDH by 100066N and 102644N. **(C)** Quantification of panel B, *n* = 3. **(D)** UDP-Glo assays were performed using 250 nM NleB1, in 125 mM Tris pH 7.4, 25 mM MnCl_2_, 2.5 mM DTT, and 100 μM UDP-GlcNAc in the presence of inhibitor concentrations ranging from 1 nM to 500 μM. **(E)** Western blot analysis of the inhibition of SseK2 glycosylation of FADD by 100066N and 102644N.

An average Z′ Score of 0.88 ± 0.05 was obtained across all 15 plate assays in the pilot screen for NleB1 inhibitors (Figure 1C). Compounds were tested in a concentration dose-response (10–160 μM) assay. Of all the compounds that inhibited the primary assay at single concentration, 80% of the hits inhibited NleB1 in a dose-responsive manner. Compounds (*n* = 52) that inhibited NleB1to >3 standard deviations ± plate median were scored as hits in the assay (hit rate of 1%; Supplemental Table 1). The two most potent compounds (100066N and 102644N) were resynthesized as fresh powders for further study (Figure 2A). 100066N is a flavone analog that has been previously synthesized (Ahn et al., 2007). 102644N is a substituted isoxazole whose synthesis has also been described (Waldo et al., 2008). Compound 104108N, which was not identified as a hit in our HTS assay, was used as a negative control in some subsequent experiments.

### *In vitro* Glycosylation Assays

We performed secondary screens to evaluate the ability of these compounds to inhibit NleB1 and SseK1 glycosylation of the human GAPDH protein substrate (Gao et al., 2013, 2016; El Qaidi et al., 2017). 100066N and 102644N were both active against both NleB1 and SseK1 (Figures 2B,C). We corroborated these data by quantifying UDP liberation in a UDP-Glo assay as a function of inhibitor concentration. Both 100066N and 102644N inhibited NleB1 activity in a concentration dependent manner, whereas compound 104108N, the negative control, did not inhibit NleB1 (Figure 2D). SseK2 glycosylates the human FADD protein (El Qaidi et al., 2017). We also tested the inhibitory effect of 100066N and 102644N on SseK2 and found that these compounds both inhibited FADD glycosylation by SseK2 in a concentration-dependent manner (Figure 2E).

### Cell Culture Assays

**Figure 3:**
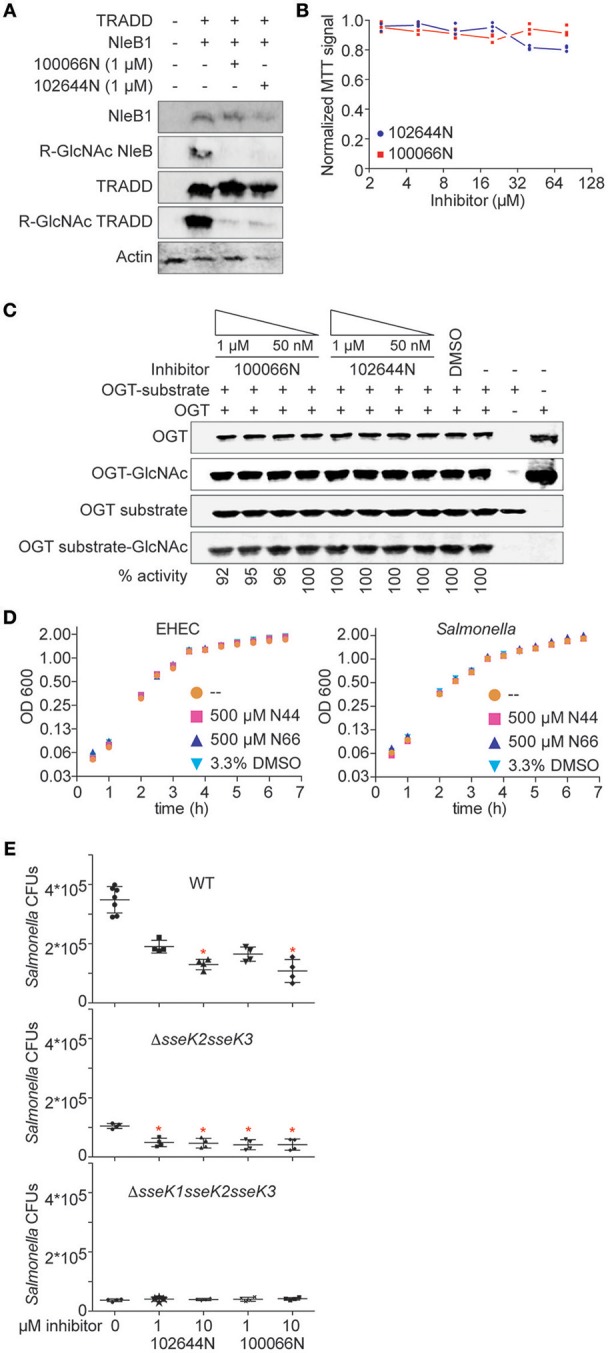
Cellular assays. **(A)** Western blot analysis of the inhibition of NleB1 glycosylation of TRADD by 100066N and 102644N in HEK293T cells. **(B)** MTT assays. Quantification of normalized MTT signal intensities as a function of 100066N and 102644N concentrations added to HEK293T cells for 24 h. **(C)** OGT assays. OGT was incubated with a recombinant OGT-peptide substrate in the presence of 100066N and 102644N and then subjected to immunoblotting using an anti-O-GlcNAc monoclonal antibody. **(D)** Bacterial growth assays. EHEC (left) and *Salmonella* (right) were cultured in the presence of the indicated compounds and growth was monitored as a function of time. **(E)** 100066N and 102644N reduce *Salmonella* replication in macrophages. Inhibitors (1 or 10 μM) were added to RAW 264.7 cells for 1 h prior to infection with 10^6^ CFUs of the indicated *Salmonella* strains. Colony counts were enumerated 24 h later. Asterisks indicate a significant difference (*p* < 0.05) from samples lacking inhibitors, as determined using one-way analysis of variance (ANOVA) with Dunnett's multiple comparisons tests.

NleB1 glycosylates human TRADD on R235, thereby blocking death domain interactions and disrupting tumor necrosis factor signaling (Li et al., 2013). We next assessed whether 100066N and/or 102644N would be effective in inhibiting NleB1 activity in mammalian cells. We co-transfected HEK293 cells with NleB1 and TRADD expression plasmids in the presence or absence of these compounds and then performed immunoblotting assays to measure TRADD glycosylation. We observed that both compounds, when provided at 1 μM concentration to HEK293T cells, were effective in reducing the extent of TRADD glycosylation by NleB1 (Figure 3A). Neither inhibitor was significantly toxic to mammalian cells, as inferred from performing 3-(4,5-dimethylthiazol-2-yl)-2,5-diphenyltetrazolium bromide (MTT) assays as a function of inhibitor concentration (Figure 3B).

The human *O*-linked *N*-acetylglucosaminyltransferase (OGT) is an essential serine/threonine *N*-acetylglucosamine (*O*-GlcNAc) transferase that maintains protein glycosylation homeostasis (Zhao et al., 2018). Deletion of OGT is lethal and deficiencies in OGT activity are associated with neurodegenerative diseases and gut inflammation (Zheng et al., 2016; Zhao et al., 2018). To consider the future use of 100066N and 102644N as potential therapeutics, we next tested whether they also inhibit OGT. Neither 100066N nor 102644N inhibited the activity of a recombinant form of human OGT (Figure 3C). These data suggest that the two NleB1 inhibitors we initially characterized are specific to the pathogen enzymes that glycosylate arginine residues, rather than cross-react with the mammalian OGT enzyme that glycosylates Ser/Thr residues (Aquino-Gil et al., 2017).

Neither 100066N nor 102644N directly impacted the growth rates of *E. coli* O157:H7 and *Salmonella enterica* when supplied at 500 μM concentrations in bacterial cultures (Figure 3D). These data suggest that the inhibitors specifically target the NleB/SseK virulence factors, rather than act as general bacteriostatic or bactericidal agents.

Finally, we quantified the impact of these inhibitors on *Salmonella* replication in RAW264.7 cells. When 102644N and 100066N (1 or 10 μM) were added to RAW264.7 cells prior to their infection with WT *Salmonella*, we observed that both inhibitors (at 10 μM) significantly reduced the number of WT *Salmonella* 24 h after infection (Figure 3E, top). We performed similar experiments with *Salmonella* Δ*sseK2sseK3* (expressing only SseK1) and observed that both inhibitors at both 1 and 10 μM further reduced the replication of *Salmonella* Δ*sseK2sseK3* (Figure 3E, middle). We also assessed the impact of 102644N and 100066N on *Salmonella* Δ*sseK1sseK2sseK3* and found there was no further impact of these inhibitors on the already relatively-low abundance of this triple-mutant strain (Figure 3E, bottom).

## Discussion

The T3SS and its effectors are of critical importance to the virulence of many Gram-negative pathogens (Gaytan et al., 2016). Bacterial virulence is significantly decreased in T3SS-deficient strains, whereas bacterial replication is not typically affected (Nordfelth et al., 2005). Thus, chemicals targeting the T3SS or its effectors are appealing, as they are less likely to be subject to evolutionary pressures toward resistance (Gu et al., 2015; McShan and De Guzman, 2015).

We screened for and identified two small molecules, 100066N and 102644N, that are effective in inhibiting the activities of NleB1, SseK1, and SseK2. We used a relatively small compound library for these initial studies and it is likely that future HTS assays using larger compound libraries may reveal more effective compounds. It is notable that although our primary screen was designed to target only the EHEC NleB1 protein, both 100066N and 102644N showed activity against not only NleB1, but also SseK1 and SseK2.

It was promising to note that neither 100066N nor 102644N were toxic to mammalian cells, nor did they directly affect *E. coli* or *Salmonella* growth. Given the significant difference in the NleB/SseK mechanism of action as compared to OGT, it was reassuring but perhaps not surprising to observe that neither inhibitor affected the *O*-linked glycosylation of an OGT substrate. We also noted that neither 100066N nor 102644N further impacted the colonization of the *Salmonella* Δ*sseK1sseK2sseK3* triple mutant, suggesting few significant off-target effects of these compounds.

The extent to which the SseK enzymes are redundant and/or are important to survival in mouse macrophages is still unclear and some of our data are in contrast to previous studies. Initially it was concluded that effects of SseK1 and SseK2 are not evident in infections of RAW 264.7 cells in a comparison of single- and double-mutants with wild-type serovar Typhimurium SL1344 (Kujat Choy et al., 2004). Later it was realized that this strain also expresses SseK3 and these experiments were reassessed (Brown et al., 2011). While no phenotype was observed in RAW 264.7 cells, it was shown that a Δ*sseK1sseK2* double mutant was attenuated in competitive index infections of mice (Brown et al., 2011). Our experiments instead used *Salmonella enterica* ATCC 14028 and thus our observation that both Δ*sseK1sseK2* and Δ*sseK1sseK2sseK3* were attenuated in macrophages in comparison to the wild-type strain may be due to strain-dependent differences. We also failed to observe significant macrophage death in our studies.

Although a structure is available in the Protein Data Bank (PDB) for SseK3 [*6EYR*; (Esposito et al., 2018)], the lack of confirmed substrates for this effector limited our ability to assess the efficacy of 100066N and 102644N against SseK3. We previously characterized the structures of NleB2 (*5H5Y*), SseK1 (*5H60*), and SseK2 (*5H61, 5H62, 5H63*) (Park et al., 2018). We are currently attempting to generate co-crystal structures between SseK1 and 100066N and 102644N both to understand how these compounds inhibit these glycosyltransferases and to identify the residues they bind in the enzymes. We speculate that 100066N and 102644N could affect UDP-GlcNAc binding and/or hydrolysis, activation of the acceptor arginine, or affect the flexibility of the HLH domain which we have attributed to being important to the recognition of the acceptor protein substrates containing death domains (Park et al., 2018). Future approaches to study the efficacy of these inhibitors will also include derivatization of both 100066N and 102644N. Logical medicinal chemistry strategies include the exploration of substitution effects on the furan and phenyl moieties at the 3 and 5 positions of 102644N as well as bioisosteric replacement of the sulfonamide linker and determining the effect of substituents on phenyl ring for 100066N.

## Experimental Procedures

### Cloning and Cell Lines

The plasmids used in this study are listed in Table 1. Human embryonic kidney 293 cells (HEK 293) and Abelson murine leukemia virus induced, macrophage-like cells from BALB/c mice (RAW264.7) were purchased from ATCC. Both cell lines were grown in DMEM (Thermo Scientific), supplemented with 10% FBS (Atlanta Biologicals) and 100 μg/ml penicillin/streptomycin (Sigma) in 5% CO_2_. *nleB1* (EHEC EDL933), *sseK1*, and *sseK2* (*Salmonella enterica* ATCC 14028) were cloned into pET42a. A human OGT substrate peptide (KKKYPGGSTPVSSANMM) was fused to the N-terminus of GAPDH and expressed in pET28a. Human FADD was expressed in pET15a. Plasmids were transformed into *E. coli* BL21 (DE3). Protein overexpression was performed for 4 h at 37°C upon induction with 0.5 mM IPTG. His-tagged proteins were purified using nickel agarose beads (Qiagen) as described previously (El Qaidi et al., 2017). *Salmonella* mutants were constructed using lambda red recombination (Datsenko and Wanner, 2000).

**Table 1:** Plasmids used in this study.

**Description**	**Source**
GST-EHEC-NleB1	El Qaidi et al., 2017
GST-EHEC-NleB1(AAA)	Gao et al., 2013
GST-SseK1	El Qaidi et al., 2017
GST-SseK2	El Qaidi et al., 2017
HA-EHEC-NleB1	Gao et al., 2013
His-FADD	El Qaidi et al., 2017
His-GAPDH	Gao et al., 2013
His-OGT	This study
His-OGT substrate (KKKYPGGSTPVSSANMM)-GAPDH	This study
TRADD-FLAG	This study

### Protein Purification

*E. coli* BL21 (DE3) strains harboring protein expression plasmids were grown at 37°C in LB containing 50 μg/ml kanamycin until an OD600 of 0.4, at which time 0.5 mM IPTG was added for 4 h further growth. Cells were harvested using centrifugation and the pellet was resuspended in 1/40 culture volume of lysis buffer (50 mM sodium phosphate NaH_2_PO_4_ pH 8.0, 0.5 mg/ml lysozyme). The suspension was incubated on ice for 30 min with occasional shaking and then an equal volume of 50 mM sodium phosphate pH 8.0, 2 M NaCl, 8 mM imidazole, 20% glycerol, 2% Triton X-100, was added for an additional 30 min of incubation. Cell lysates were sonicated, centrifuged, and then the resultant supernatant was added to 2 ml Ni-NTA resin (Qiagen) for 1 h of rotation at 4°C. The mixture was loaded on a Poly-Prep Chromatography Column (Bio-Rad) and washed with 10 ml of 50 mM sodium phosphate pH 8.0, 600 mM NaCl, 60 mM imidazole, 10% glycerol. Proteins were eluted in 2 ml 50 mM sodium phosphate pH 8.0, 600 mM NaCl, 250 mM imidazole, 10% glycerol and dialyzed into the same buffer lacking imidazole overnight at 4°C. We note that some of the proteins appeared to show cleavage of the epitope tag after purification and other proteins formed higher molecular weight aggregates when resolved using SDS-PAGE.

### HTS Assay Optimization

The glycosyltransferase activity of NleB1 was tested using the luminescence based UDP-Glo™ Glycosyltransferase Assay (Promega) according to the manufacturer's instructions. The optimized assay was performed by incubating purified NleB1 (150 nM) with UDP-GlcNAc (300 μM) for 2 h at 30°C, followed by the addition of UDP detection reagent to covert UDP to ATP, which is then utilized by luciferase to generate light in proportion to the UDP concentration. The luminescence was read using a BioTek Synergy Neo (BioTek Inc.). The assay was optimized for buffer components (MgCl_2_, NaCl), linearity of the assay, the concentration ranges of NleB1 and UDP-GlcNAc, optimal temperature, and the Km of NleB1 for UDP-GlcNAc.

### Pilot Library Screening

The optimized assay was used to screen a KU Legacy collection of 5,160 diversity compounds synthesized by The Chemical Methodologies and Library Development (CMLD) Center. Assays were performed in buffer containing 50 mM Tris pH 7.5, 100 mM NaCl, 1 mM DTT, 10 mM MgCl_2_ and 0.5% DMSO. NleB1 (150 nM) was pre-incubated for 30 min in 384 well-microplates, containing CMLD compounds (20 μM) transferred acoustically using ECHO555 (Labcyte Inc). Luminescence was read 2 h after the addition of the UDP detection reagent. Each screening plate contained 16 wells of minimum signal (no enzyme, substrate only in 0.5% DMSO, for average and standard deviation calculations, SD min) and 16 wells of maximum signal (enzyme + 0.5% DMSO + Substrate, for average and standard deviation calculations, max). The distribution of controls across each plate was used to calculate Z′ scores, using the equation z′ = $\left(1-\frac{3SDmin+3SDmax}{Average\;max-Average\;min}\right)$. The percent inhibition was normalized to the controls and compounds that inhibited the assay to >3 standard deviations plus the plate median were identified as hits.

### Glycosylation Assays

*In vitro* glycosylation assays were performed as described previously (El Qaidi et al., 2017). Briefly, 200 nM of enzyme (NleB1, SseK1, or SseK2) was incubated with 1 μM of GAPDH or FADD in the presence or absence of the studied inhibitors in buffer containing 50 mM Tris-HCl pH 7.4, 1 mM UDP-GlcNAc, 10 mM MnCl_2_, and 1 mM DTT. After 2 h incubation at RT, samples were subjected to western blotting using an anti-R-GlcNAc monoclonal antibody (Abcam). Assays with OGT were performed in buffer containing 25 mM Tris-HCl pH 7.4, 1 mM UDP-GlcNAc, 12.5 mM MgCl_2_, and 1 mM DTT. After 4 h of incubation at RT, samples were subjected to western blotting using an anti-O-GlcNAc monoclonal antibody (Millipore). Signal intensities were quantified using Li-COR Image Studio software and inhibition was calculated by quantifying the relative reduction in substrate glycosylation after normalization to the effect of DMSO.

### UDP-Glo Assays

UDP-Glo luminescence assays were performed in 96 well-microplates, containing 250 nM NleB1 in 125 mM Tris pH 7.4, 25 mM MnCl_2_, 2.5 mM DTT, and 100 μM UDP-GlcNAc in the presence of inhibitor concentrations ranging from 1 nM to 500 μM. Reactions were incubated for 2 h at 30°C and then developed and quantified using a FLUOstar microplate reader (BMG Labtech).

### Transfections

HEK293T cells were co-transfected with NleB1-HA and TRADD-FLAG plasmids in the presence or the absence of inhibitors. At 24 h post-transfection, cells were washed with PBS, suspended in SDS sample buffer, boiled for 10 min, and immunoblotted with an anti-R-GlcNAc monoclonal antibody (Abcam).

### MTT Assays

MTT assays were performed as specified by Millipore using HEK 293 cells in the presence of 2-fold serial dilutions of 100066N or 102644N. Formazan absorbance was measured using an Epoch Microplate Spectrophotometer (BioTek).

### Macrophage Infections

Macrophage infection assays were performed to measure the intracellular invasion of bacteria in the presence of 100066N or 102644N. RAW264.7 cells were seeded at 1 x 10^5^ cells/well in 24-well plates and inhibitors were added at 1 or 10 μM final concentrations 1 h before infection. Bacterial cultures were grown overnight and 10^6^ CFUs were added to each well for 30 min. Cells were then incubated in medium containing 100 μg/mL gentamicin for 1 h, and then 10 μg/mL gentamicin for an additional 23 h. Bacteria were released from RAW264.7 cells using 1% saponin, diluted in PBS, and plated for colony counts.

### Statistical Analyses

Data were analyzed statistically using one-way analysis of variance (ANOVA) with Dunnett's multiple comparisons tests. *P*-values < 0.05 were considered significant.

## Author Contributions

PH conceived and coordinated the study and wrote the paper. SE, CZ, PM, and AR performed and analyzed the experiments. PKM, DR, and CP assisted with chemical synthesis. All authors reviewed the results and approved the final version of the manuscript.

### Conflict of Interest Statement

The authors declare that the research was conducted in the absence of any commercial or financial relationships that could be construed as a potential conflict of interest.
