# Supplementary material for: High-Throughput Screening for Bacterial Glycosyltransferase Inhibitors
Source: Front Cell Infect Microbiol. 2018 Dec 18;8:435. doi: 10.3389/fcimb.2018.00435 (PMC6305410; doi:10.3389/fcimb.2018.00435)
Supplement: Supplementary file 1 [file Table_1.DOCX]

**Supplemental Table 1. Active compound subset.** The 52 compounds that inhibited NleB1 activity to greater than 3 standard deviations + the plate median are shown. Chemical structures are designated using smiles string notation, followed by the % NleB1 inhibition as a function of compound concentration.

|  |  | **% NleB1 inhibition**  **(µM compound)** | | | | |
| --- | --- | --- | --- | --- | --- | --- |
| **ID** | **Smiles String** | **10** | **20** | **40** | **80** | **160** |
| KUC104101N | O=C(C1=CC=C(O)C=C1)CN2N=NC(COC3=CC=C(NC(C)=O)C=C3)=C2 | 48.1 | 59.3 | 74.6 | 91.3 | 98.1 |
| KUC102644N | O=C(O)C1=C2C(C=CC=C2)=NC(C(C=C3)=CC=C3C4=C(C5=CC=CC=C5)ON=C4C6=CC=CO6)=C1 | 50.1 | 58.4 | 75.0 | 89.4 | 97.2 |
| KUC100066N | Oc1ccc(NS(=O)(c2cc(Cl)cc(Cl)c2O)=O)c(OC(c3c(Cl)cccc3)=C4)c1C4=O | 43.0 | 71.1 | 84.8 | 96.0 | 96.8 |
| KUC101994N | O=S1(C2=CC=C(C=C2[C@@H](N1CCCCCCCCCCCC)CC(O)=O)C(F)(F)F)=O | 40.6 | 48.0 | 60.1 | 80.4 | 94.3 |
| KUC104114N | O=C(C1=CC=C(O)C=C1)CN2N=NC(COC3=CC=CC(C=O)=C3)=C2 | 31.8 | 42.3 | 57.2 | 76.5 | 92.0 |
| KUC104088N | O=C(C1=CC=C(O)C=C1)CN2N=NC(COC3=C(F)C=C(C(C)=O)C=C3)=C2 | 45.4 | 47.7 | 59.8 | 76.5 | 92.0 |
| KUC104108N | O=C(C1=CC=C(O)C=C1)CN2N=NC(COC3=CC=C(C(CC)=O)C=C3)=C2 | 21.0 | 35.3 | 49.4 | 67.7 | 88.0 |
| KUC104090N | O=C(C1=CC=C(O)C=C1)CN2N=NC(COC3=C(C)C=CC=C3C)=C2 | 36.8 | 44.9 | 53.2 | 69.2 | 85.8 |
| KUC104109N | O=C(C1=CC=C(O)C=C1)CN2N=NC(COC3=CC=C(OC)C=C3)=C2 | 35.4 | 43.6 | 53.9 | 67.3 | 82.9 |
| KUC103319N | O=S1(N(CC2=CC(F)=CC=C2)C3=CC=C(Br)C=C3C(C1)=O)=O | 32.5 | 37.2 | 49.0 | 68.8 | 81.7 |
| KUC104092N | O=C(C1=CC=C(O)C=C1)CN2N=NC(COC3=C(F)C(F)=CC(F)=C3F)=C2 | 39.5 | 42.1 | 57.8 | 67.2 | 80.6 |
| KUC105137N | COC1=CC=C(C2=CC=C(C=C2)CN3C(C(N(CC4=CN(N=N4)CCC5OCCO5)CCS3(=O)=O)=O)C(C)C)C=N1 | 13.7 | 25.7 | 41.5 | 64.5 | 79.7 |
| KUC104115N | O=C([C@@H](C)C1=CC=C(C=C(OC)C=C2)C2=C1)OCC3=CN(CC(C4=CC=C(O)C=C4)=O)N=N3 | 40.2 | 44.0 | 48.2 | 61.4 | 78.6 |
| KUC110967N | C[C@H]1CC[C@@]2(N(CCCOC(C3=CC=CC=C3)=O)C(CC2)=O)CC1 | 45.2 | 53.8 | 62.0 | 71.4 | 78.5 |
| KUC104085N | O=C(C1=CC=C(O)C=C1)CN2N=NC(COC3=CC=CC=C3C(F)(F)F)=C2 | 36.1 | 4538 | 51.8 | 63.6 | 78.4 |
| KUC104099N | O=C(C1=CC=C(O)C=C1)CN2N=NC(COC3=CC=C(C(CCC)=O)C=C3)=C2 | 32.9 | 42.2 | 45.2 | 62.5 | 77.5 |
| KUC103038N | O=C1CCC[C@]2([H])CC3=NC4=C(C=C(O)C=C4)C=C3[C@@]5([H])[C@]2([H])N1CC5 | 26.7 | 35.2 | 34.4 | 47.9 | 76.1 |
| KUC105133N | COCOCCN1C=C(N=N1)CN2CCS(=O)(N(C(C2=O)C(C)C)CC3=CC=C(C4=CC=C(N=C4)OC)C=C3)=O | 30.4 | 40.4 | 45.5 | 62.2 | 75.8 |
| KUC104076N | CC(C1=CC(OC)=C(OCC2=CN(CC(C3=CC=C(O)C=C3)=O)N=N2)C=C1)=O | 32.0 | 44.9 | 45.1 | 61.1 | 75.5 |
| KUC105149N | COCOCCN1C=C(N=N1)CN2CCS(=O)(N(C(C2=O)CC(C)C)CC3=CC=C(C4=CC=C(N=C4)F)C=C3)=O | 34.4 | 38.8 | 42.6 | 55.9 | 69.5 |
| KUC108511N | O=S1(C2=CC=C(N3CC[C@@H](O)C3)C=C2O[C@H](COCC4=CC=CC=C4)CN1[C@@H](C5=CC=CC=C5)C)=O | 33.8 | 33.0 | 29.9 | 49.8 | 63.7 |
| KUC100143N | O=C1CCN(C(C2=CC=CC(C)=C2)=O)CCN1[C@H](COCC3=CC=C(Cl)C=C3)CC4=CC=CC=C4 | 35.9 | 38.0 | 39.2 | 42.0 | 63.3 |
| KUC101437N | OC(O1)(c2ccccc2)CC(c3ccc(OC)cc3)c4c1c(C)ccc4C | 10.8 | 13.9 | 23.3 | 39.1 | 57.3 |
| KUC106008N | O=S1(C2=C(OC3=C(OC)C=C(F)C=C3)C=CC=C2OC(COCC4=CC=CC=C4)CN1CCCC)=O | 21.4 | 22.4 | 20.7 | 31.6 | 55.9 |
| KUC105151N | CC(CC1N(S(=O)(CCN(C1=O)CC2=CN(N=N2)CCC3OCCCO3)=O)CC4=CC=C(C5=CC=C(N=C5)F)C=C4)C | 0.6 | 15.1 | 31.9 | 50.8 | 53.0 |
| KUC105040N | CCCCCC(N1C[C@H](N2C=C(N=N2)COC(C3=CC=CC=C3)=O)[C@H](C[C@@H]1C4=CC=C(C=C4)Cl)O)=O | 9.1 | 14.5 | 18.9 | 40.4 | 50.7 |
| KUC104086N | O=C(C1=CC=C(O)C=C1)CN2N=NC(COC3=CC=CC([N+]([O-])=O)=C3)=C2 | 24.5 | 24.5 | 34.2 | 32.8 | 50.5 |
| KUC102262N | O=S1(C2=CC(/C=C/C(O)=O)=CC=C2[C@@H](N1C3=CC=CC(OC)=C3)CC(O)=O)=O | 27.6 | 34.2 | 35.9 | 41.7 | 46.5 |
| KUC102739N | O=S1(CCOC[C@H](C2=CC=CC=C2)N1CC3=CC(Cl)=C(Cl)C=C3)=O | 16.3 | 16.2 | 25.3 | 35.0 | 45.5 |
| KUC101184N | O=C(CN(Cc1ccccc1)S2(=O)=O)c3c2cccc3 | 2.9 | 18.5 | 23.8 | 33.3 | 44.7 |
| KUC104889N | CC1N(S(=O)(CCN(C1=O)CC2=CN(N=N2)CCC3OCCCO3)=O)CC4=CC=C(C5=CC=CC(N(C)C)=C5)C=C4 | 26.6 | 28.9 | 29.5 | 35.9 | 44.5 |
| KUC101459N | COc1cc(OC)c(C(c2ccc(OC)cc2)CCN3CCOCC3)c(O4)c1C(c5ccc(OC)cc5)CC4=O | 14.9 | 26.2 | 9.4 | 18.8 | 44.1 |
| KUC107029N | O=S1(C=C[C@H](N1C(C2=CC=C(C=C2)[N+]([O-])=O)=O)COCC3=CC=CC=C3)=O | 30.6 | 41.5 | 43.4 | 48.6 | 43.4 |
| KUC111570N | O=C(C1=CC=C(F)C=C1)N2CCCC3(CC(C4=CC(Cl)=CC=C4)=CO3)C2 | 3.2 | 3.8 | 5.2 | 24.2 | 42.6 |
| KUC101907N | FC1=CC([C@@H](N2CCCCCCCC)CC(OC)=O)=C(C=C1)S2(=O)=O | 23.0 | 22.1 | 22.4 | 29.9 | 42.2 |
| KUC111139N | O=S1(C2=CC=C(N3[C@H](C)CCC3)C=C2O[C@]4([H])CCN1C4)=O | 32.7 | 34.1 | 35.7 | 38.7 | 40.7 |
| KUC102830N | O=C1N(CCSC2=CC=CC=C2)CCC(NC(OCC3=CC=CC=C3)=O)CC1 | 16.9 | 20.9 | 21.9 | 25.7 | 38.2 |
| KUC103993N | O=S(C1=C(F)C=CC(Cl)=C1)(N[C@H](C)[C@H](O)C2=CC=CC=C2)=O | 27.5 | 31.8 | 31.8 | 34.5 | 37.0 |
| KUC100988N | O=C1N(CCOCC2=CC=C(Cl)C=C2)CCN(S(C3=CC=CC=C3)(=O)=O)CC1 | 31.9 | 14.5 | 26.3 | 28.3 | 36.0 |
| KUC112275N | O=C1CCC[C@@]2([H])[C@@H](CC3=CC=CC=C3)[C@@H](OC(OC4=CC=C([N+]([O-])=O)C=C4)=O)CC[C@@]25N1CCC5 | 2.6 | 5.6 | 11.0 | 24.2 | 34.9 |
| KUC101608N | O=C1N(Cc2ccc(F)cc2)C(c3ccc(OC)cc3)\C=C/CN(C(CCCCCC)=O)[C@H]1Cc4ccccc4 | 15.6 | 17.3 | 23.2 | 30.3 | 32.5 |
| KUC112136N | O=C1CCC[C@@]2([H])[C@@H](CCC)[C@@H](OC(NCC3=CC=C(OC)C=C3)=O)CC[C@@]24N1CCC4 | 15.7 | 22.0 | 20.8 | 28.5 | 30.5 |
| KUC109525N | O=S1(N(C2CCCC2)C(N3CC4=CC=C(C)C=C4C3C1)=O)=O | 26.3 | 31.5 | 20.9 | 19.5 | 29.3 |
| KUC102268N | O=S1(C2=CC(/C=C/C(OC)=O)=CC=C2[C@@H](N1CCCCCCCC)CC(OC)=O)=O | 25.3 | 20.8 | 27.1 | 33.3 | 27.9 |
| KUC103195N | C[C@H](NC1=O)[C@@H](O1)C2=CC=CO2 | 23.8 | 25.6 | 28.1 | 33.5 | 23.9 |
| KUC108117N | O=S1(CC(N2CCN(CCN(C)C)CC2)CN1CC3=CN(CC4=CC=C(F)C=C4)N=N3)=O | 5.6 | 23.7 | 25.1 | 21.8 | 21.8 |
| KUC102485N | O=C1N(CCC#N)CCN(CC(C)C)CC1 | 16.0 | 23.1 | 17.6 | 19.0 | 20.8 |
| KUC111012N | CC1=CC=C([C@@H]2CC[C@]3(N(CCOC(NC4=CC=CC(C#N)=C4)=O)C(CC3)=O)CC2)C=C1 | 17.3 | 25.1 | 22.4 | 20.7 | 20.5 |
| KUC108236N | O=S1(CC(N2CCN(CC3=C(F)C=CC=C3)CC2)CN1CC4=CN(CC5=CC=CC(Cl)=C5)N=N4)=O | 13.5 | 26.7 | 13.1 | 12.7 | 18.4 |
| KUC108133N | O=S1(CC(N2CCN(C3CCN(C)CC3)CC2)CN1CC4=CN(CC5=CC=CC(Cl)=C5)N=N4)=O | 4.1 | 18.2 | 22.3 | 18.0 | 18.2 |
| KUC103094N | O=S(N(CC[C@H]1O)CC=C)(C1=C)=O | 19.8 | 23.8 | 19.2 | 18.2 | 18.0 |
| KUC110604N | O=S1(C2=CC=CC=C2C(N(CC3=CC=CC=C3)N=N4)=C4CN1[C@H](C(OC)=O)C(C)C)=O | 4.8 | 15.1 | 17.9 | 21.1 | 17.9 |
